# Always Gamble on an Empty Stomach: Hunger Is Associated with Advantageous Decision Making

**DOI:** 10.1371/journal.pone.0111081

**Published:** 2014-10-23

**Authors:** Denise de Ridder, Floor Kroese, Marieke Adriaanse, Catharine Evers

**Affiliations:** Department of Clinical and Health Psychology, Utrecht University, Utrecht, The Netherlands; Centre national de la recherche scientifique, France

## Abstract

Three experimental studies examined the counterintuitive hypothesis that hunger improves strategic decision making, arguing that people in a hot state are better able to make favorable decisions involving uncertain outcomes. Studies 1 and 2 demonstrated that participants with more hunger or greater appetite made more advantageous choices in the Iowa Gambling Task compared to sated participants or participants with a smaller appetite. Study 3 revealed that hungry participants were better able to appreciate future big rewards in a delay discounting task; and that, in spite of their perception of increased rewarding value of both food and monetary objects, hungry participants were not more inclined to take risks to get the object of their desire. Together, these studies for the first time provide evidence that hot states improve decision making under uncertain conditions, challenging the conventional conception of the detrimental role of impulsivity in decision making.

## Introduction

In their daily lives, people are frequently confronted with self-control dilemmas requiring them to choose between an immediate but small reward or a larger reward in the long run. [1] Opting for a small reward when a bigger one is available can be regarded as self-control failure, even when the bigger one is delayed. Yet, many people tend to engage in this kind of disadvantageous choices, such as weight watchers who prefer a high caloric muffin for breakfast over a slim waist or business men preferring a night out at the casino over preparing next day's meeting. Hot states like emotions or visceral drives have a bad reputation of compromising such self-control dilemmas by making people less patient to wait for the long-term benefits. [2], [3].

Indeed, an overwhelming amount of evidence exists indicating that people become more impulsive and opt for immediate gratification of their desires when they are emotional, hungry, sexually aroused or otherwise in a hot state. For example, sexually aroused people engage in more impulsive decision making about sexual encounters, even when aware of the potential negative consequences. [4] Also, hungry people become more wanting of food [5] and tend to forget about their weight goals [6]. These findings make sense when considering that hot states in general, and visceral drives in particular, are adaptive physiological states that increase the motivation to satisfy important immediate (physical) needs, such as drinking water when thirsty. [2] However, these urges become problematic when they are in conflict with a long term-goal, such as indulging in food when someone has a goal of weight watching or engaging in incidental sexual encounters when one has the goal of being faithful in a marital relationship. Moreover, and potentially even more problematic, visceral states do not only affect motivation for rewards corresponding with the drive (e.g., getting food when hungry) but also may generalize to unrelated rewarding behaviors, such as when people report a stronger desire for money when hungry [7] or become more impulsive when sexually aroused [8]. There thus seems to be consensus that visceral drives and other hot states make people myopic for the future' and hurt their long-term interests. [9].

Despite the apparent evidence that hot states compromise advantageous decision making, most studies so far have examined decisions about satisfaction of immediate needs in simple decision contexts where the potential long-term consequences are relatively straightforward. In contrast, there is initial evidence suggesting that hot states may not compromise but rather facilitate advantageous decision making when these decisions are complex and long-term outcomes are uncertain, such as when delayed benefits are involved. In this kind of situations one cannot always rationally deliberate on multiple alternatives and reflect on their future consequences; other sources of information, such as intuitions [10], [11] and emotions [12], [13] are required. According to Damasio and coworkers [14], in situations of uncertainty or complexity, emotional responses may provide valuable information about the potential consequences of a behavioral option, and enable faster and experience-driven decision making by steering attention towards the outcomes to which a given action may lead. To illustrate, it has been demonstrated that people with deficits in brain areas involved in the processing of emotions (ventromedial prefrontal cortex) perform worse on complex decision-making tasks. [9] Relying on one's gut feeling, as normal people without emotion processing deficits typically do, thus may favor rather than compromise decisions with uncertain long-term benefits. [14] From these findings, it can be inferred that hot states are crucial in promoting advantageous decision making in contexts that do not allow for explicit deliberation about the long-term consequences of these decisions.

Thus far, direct experimental evidence for the notion that hot states may support advantageous decisions with uncertain outcomes is lacking. There is only indirect evidence showing that people with impulsive disorders who suffer from deficits in emotional processing perform poorly on complex decision tasks. [15], [16], [17] Studies examining the straightforward benefits of hot states - rather than the disadvantages of not being able to use one's emotions in complex decision making - are lacking. Moreover, there are no studies that examine the causal effect of hot states on complex decision making in normal samples, precluding conclusions about the assumed advantages of hot states in uncertain conditions. Findings from a correlational study in a non-clinical sample, however, lend initial support to our counterintuitive hypothesis, showing that stock investors who experienced more intense feelings achieved a better decision-making performance. [18] In the present study, we explore the notion that people who have an inclination to act on impulse because of being in a hot state, are capable of decisions that increase their maximum benefit when complex decisions with uncertain outcomes are involved. Specifically, we examine the novel idea that the hot state of being hungry may leave people to rely more on their gut feeling and that this does not necessarily lead to bad decisions but may rather foster advantageous decision making when these decisions are too complex to explicitly deliberate upon their consequences. While there is until now no direct evidence suggesting that people in hot states may benefit from their impulsive inclinations, this idea aligns well with recent literature challenging the sharp distinction that has been made in dual systems theories of self-regulation between cool and hot systems. [19] Whereas traditionally it is assumed that the cool system is responsible for ‘good’ behaviors and the hot system produces ‘bad’ behaviors, it is now acknowledged that sometimes impulsive states can generate adaptive behavior as well. [20] These new insights provide a reason to explore the idea that hot states may benefit rather than harm decision making.

### Overview of studies

In three studies we tested the hypothesis that hot states promote advantageous decision with uncertain long-term outcomes by manipulating hunger or appetite as a typical hot state that is characterized by a strong visceral drive. [2], [5] As a dependent variable (Studies 1 and 2) we used the Iowa Gambling Task (IGT) to assess performance on a complex decision task with uncertain outcomes. [21] The IGT involves a valid simulation of decision making about immediate gratification and long-term monetary benefits under uncertain conditions that have some resemblance with the difficulties of real-life decision making. Although the IGT is a lab task that cannot capture all complexities of decision making in real life situations, it is generally agreed that one has to rely to some extent on one’s feelings and hunches in order to be successful on the IGT, thus providing evidence that the IGT is a valid task for assessing decision making under uncertain conditions. [14] Several authors have conceptually classified the IGT as a distinct measure of complex decision making without having the possibility of explicit deliberation [22] and relying on intuition [23]. Alternatively, we employed a delay discounting task (Study 3) to assess whether a hungry state would also lead to a preference of long-term profit over immediate benefits. [24] The delay discounting task is conceptually similar to the IGT insofar it involves an extended series of complex decisions about short-term vs. long-term reward which make it impossible to rationally calculate the overall favorable outcome.

Manipulation of a hot state in one domain (hunger) and examining its effects in another (monetary) may demonstrate that the advantages of a hot state transcend the particular behavioral domain and thus provides a particularly strong test of our hypothesis that hot states benefit decision making under uncertainty. In Study 1, we examined how the hot state of hunger, compared to being sated, affects complex decision making under uncertain conditions as assessed by IGT performance. Study 2 investigated whether an alternative measure of a hot state (appetite) would affect this kind of decision making (also assessed by IGT performance). The main goal of Study 3 was to replicate findings from the first two studies employing an alternative measure of complex decision making (delay discounting task). Study 3 also examined to what extent a hot state (hunger) affected risk taking (insensitivity to future consequences) and reward perception (being driven by immediate reward) as the two main components of decision making under uncertainty. [15] We hypothesize that hot states will lead to an increased perception of reward but does not necessarily result in taking more risks to obtain that reward. This hypothesis challenges common explanations of decision making in hot states that would predict an increased willingness to take risk. [3] Additionally, Study 3 tested whether the effect of hunger on complex decision making concerning money was an artifact of decreased motivation for monetary rewards resulting from being preoccupied with food. In all three studies, we accounted for individual differences in impulsivity as assessed by different constructs (impulsivity, self-control, Behavioral Inhibition System/Behavioral Activation System), as these constructs share conceptual similarities but are slightly distinct. As such, they allow for a better exploration of how trait impulsivity may affect the impact of hot states on decision making. [25].

## Study 1

The objective of this study is to determine whether people in a hot state who are hungry perform better on a complex decision task with uncertain outcomes (IGT) than people in a cold state who are sated.

### Method

#### Participants

Thirty normal weight to slightly overweight (Body Mass Index [BMI]: *M* = 22.08, *SD* = 2.42; range 18.42–25.95) university students (nine men, 21 women; mean age 21.97 years) participated in exchange for money (3€) or course credit.

#### Ethics statement

The study was conducted in accordance with the ethical standards described by the Medical Research Involving Human Subjects Act (WMO). [26] This Act exempts research on healthy human subjects from review for as long as it does not involve any invasion of participants’ integrity. To determine whether our study fell within the remit of the Act, the study procedure was assessed by the Review Board of the Faculty of Social Sciences of Utrecht University, consistent with the Faculty protocol. The Board rated the study as not being invasive of the participants’ integrity, and hence not subject to WMO. Consequently, no formal ethical approval was required according to Dutch national standards. Written consent was required from each participant prior to participation.

### Procedure and Materials

#### Hunger manipulation

All participants were instructed to refrain from eating and drinking (except water) from 11 pm in the evening prior to their session. Upon arriving at the lab the next morning (in two time slots, either at 8.30 AM or 9.15 AM), they were randomly assigned to either the cool (sated) condition in which the IGT was administered after consuming breakfast, or to the hot (hunger) condition in which the IGT was completed before breakfast consumption. Participants were instructed that they were free to eat and drink as much they wanted from the breakfast that was served after having consumed 200 ml of thick yoghurt to assure a minimal level of satiation. [27] Before administration of the IGT participants self-reported hunger was assessed on a 5-point scale ranging from 1, *not at all*, to 5, *very much*.

#### Assessment of complex decision making

Complex decision making under uncertain conditions was assessed by means of a computerized version of the IGT. [28] Participants were presented with four decks of cards (A–D) and were told that the task requires a series of card selections, one card at the time, from any of the four decks, until they were told to stop. Card selections were made by a mouse click on the chosen card. They were not told how many choices had to be made, but the task stopped after 100 trials. The instructions and the win and loss schedules were similar to those used by Bechara et al. [21] After choice A or B, the participant received €100, after choice C and D they received €50. However, some cards from decks A–D required the participant to pay a penalty, often higher than the amount of money received on that card. In deck A the penalties varied between €150 and €350. In deck B there was one penalty of €1250. In deck C the penalties varies between €25 and €75. In deck D there was one penalty of €250. Thus, in the high-paying decks (A–B) the penalties were higher as well, resulting in a negative balance of €250 per 10 trials for both decks. In the low-paying decks (C–D), the modest penalties resulted in a net gain of €250 per 10 trials for both decks. This means that decks A and B were disadvantageous in the long run, whereas deck C and D were advantageous in the long run. The number of card selections from advantageous decks in the final 60 trials was used as the dependent measure, as there is evidence that inhibitory processes become more important during the latter half of the task when participants are more aware of the risk status of each deck. [29] Analyses using the full set of 100 trials yielded similar significant results (not reported).

#### Other variables

To rule out the possibility that any differences between conditions on IGT performance were due do individual differences in impulsivity, the 30 item Barratt Impulsivity Scale (e.g., “I do things without thinking”) was administered, employing a 5-point scale ranging from 1, *not at all*, to 5, *very much*. [30] The scale had good internal consistency, Cronbach's alpha = .76. Finally, demographic information was collected by asking participants about gender, age, weight and length. The latter two variables were used to compute BMI (weight/height * height). BMI was registered to account for its potential to affect hunger ratings of the participants.

### Results

#### Randomization check

A series of Analyses of Variance (ANOVAs) demonstrated the absence of differences in gender, age, BMI, or impulsivity across conditions, all *F*s <1, indicating successful randomization.

#### Manipulation check

Testing the validity of the hunger manipulation, an ANOVA with condition as the independent variable and hunger as the dependent variable yielded a significant effect, *F*(1, 27) = 47.23, *p*<.001, p*η* ^2^ = .64: participants in the hunger condition reported more hunger (*M* = 4.14, *SD* = .77) than those in the sated condition (*M* = 1.87, *SD* = .99).

#### Main analysis

Before testing our hypothesis that a hot state (being hungry) would result in more advantageous decision making, we examined whether BMI and the Barratt Impulsivity Scale correlated with IGT performance. The correlation of the Barratt Impulsivity Scale with IGT performance was not significant (*r* = .02, *p* = .88); this scale was therefore not included as a covariate. The correlation of BMI with IGT performance was significant (*r* = .38, *p*<.05); BMI was therefore included as a covariate. Next, an ANCOVA was conducted with condition as the independent variable, number of card selections from advantageous decks as the dependent variable, and BMI as a covariate. A significant effect of condition was found, *F*(1, 25) = 4.52, *p*<.05, p*η* ^2^ = .15: hungry participants selected more cards from the advantageous decks (*M* = 33.36, *SD* = 12.48) than did sated participants (*M* = 25.86; *SD* = 12.16). BMI was a significant covariate, *F* (1, 25) = 6.33, *p*<.05, p*η* ^2^ = .20 (Table 1).

**Table 1:** Study 1: Card selections from advantageous decks (final 60 trials)*.

	M (SD)
Sated	25.86 (12.16)
Hungry	33.36 (12.48)

*BMI is a significant covariate.

### Discussion

Study 1 shows that people who were hungry because of having fasted overnight performed better on a complex decision task than sated people and thus provides a first piece of evidence that the hot state of hunger improves, rather than compromises, advantageous decision making. This finding is even more impressive when considering that the physiological effects of hunger have been shown to decrease cognitive performance because of having fewer cognitive resources available [31], while in our study hungry participants performed better on selecting card from advantageous decks - thus lending credit to our assumption that hungry participants performed better in this complex task with uncertain outcomes.

## Study 2

The objective of Study 2 was to replicate the findings from Study 1 by employing a more subtle manipulation of a hot state by increasing appetite for food, ruling out potentially physiological effects of hunger that may interfere with our aim to manipulate hot states.

### Method

#### Participants

Fifty female normal weight to slightly overweight (BMI: *M* = 21.11, *SD* = 2.01; range 16.22–27.34) university students (mean age 20.5 years) participated in exchange for money (3€) or course credit.

#### Ethics statement

See Study 1. All participants provided written informed consent.

### Procedure and Materials

#### Manipulation of appetite and assessment of complex decision making

Upon arrival at the lab, participants were randomly assigned to either the hot (focus on appetite for snacks) or the cool (focus on price of snacks) condition, presenting them with ten popular snacks which they had to evaluate according to appetite ('To what extent do you feel like having [snack] at this moment?' on a scale from 1, *not at all*, to 7, *very much*) or according to price ('How do you evaluate the price of [snack]?' on a scale from 1, *very cheap*, to 7, *very expensive*). After having completed the IGT (procedure similar as in Study 1), participants indicated on 5-point scales ranging from 1, *not at all*, to 5, *very much*, whether they had an appetite for snacks (mean score of three items; e.g., “I feel like a bite”; Cronbach's α = .89) and whether they were hungry (same item as in Study 1). In line with our aim to provide a subtle manipulation, these ratings were obtained after IGT completion to avoid a focus on appetite.

#### Other variables

To rule out the possibility that differences between conditions on IGT performance were due do individual differences in trait self-control, the 13 item Brief Self-Control Scale (e.g., “I am good at resisting temptations”) was administered, employing a 5-point scale from 1, *not at all*, to 5, *very much*. [32] The scale had good internal consistency, Cronbach's alpha = .75. Finally, demographic information was collected by asking participants about age, weight and length. The latter two variables were used to compute BMI.

### Results

#### Randomization check

We first tested whether there were any differences between conditions in terms of age, BMI, or trait self-control. A series of ANOVAs with condition as the independent variable and age, BMI, and self-control as the dependent variables revealed no significant effects, all *F*s <1, indicating successful randomization.

#### Manipulation check

To test whether the hot/cool manipulation was effective, an ANOVA with condition as the independent variable and appetite as the dependent variable was conducted, yielding a significant effect, *F*(1, 48) = 5.73, *p*<.05, p*η* ^2^ = .11. Participants in the hot condition reported greater appetite (*M* = 3.46, *SD* = .92) than participants in the cool condition (*M* = 2.81, *SD* = .99). There was no significant difference in hunger between conditions, *F*(1, 48) = 1.40, *p* = .24.

#### Main analysis

Before conducting the main analysis, we examined whether the Self-Control Scale was associated with IGT performance. The correlation was not significant (*r* = .05, *p* = .73). This scale was therefore not included as a covariate. We also examined whether BMI was associated with IGT performance; the correlation was not significant (*r* = .07, *p* = .65). To test our main hypothesis that a hot state would yield more advantageous decision making, an ANOVA was conducted with condition as the independent variable and number of card selections from advantageous decks of the latter 60 trials (similar as in Study 1) as the dependent variable. A significant condition effect was found, *F*(1, 48) = 6.80, *p<*.05, p*η* ^2^ = .12. Participants in the hot condition selected more cards from the advantageous decks (*M* = 28.89, *SD* = 9.01) compared to participants in the cool condition (*M* = 22.13, *SD* = 9.28). For reasons of comparing the results of Study 2 with those of Study 1, we replicated the main analysis with BMI as a covariate. This analysis yielded similar results as the analysis without BMI as a covariate, *F*(1, 47) = 6.44, *p<*.05, p*η* ^2^ = .12; the covariate was not significant (*p* = .90) (Table 2).

**Table 2:** Study 2: Card selections from advantageous decks (final 60 trials).

	M (SD)
Cool focus	22.13 (9.28)
Hot focus	28.89 (9.01)

### Discussion

Study 2 again provided evidence for our hypothesis that a hot state benefits advantageous decision making. Participants with a larger appetite were more likely to make advantageous decisions as witnessed by more choices involving small wins and small losses, regardless their level of trait self-control. However, these findings do not address the question how hot states affect the two main components involved in strategic decision making, risk taking and reward perception. Therefore, Study 3 also included separate assessments of risk taking and perceived reward value to examine our hypothesis that hunger would affect reward perception but not risk taking. To rule out the possibility that our findings were restricted to IGT performance solely, we also used an alternative task (delay discounting task) for assessing complex decision making under uncertain conditions. Additionally, we wanted to rule out the alternative explanation that a hot state like hunger benefits decision making because of being less concerned about money than about food.

## Study 3

The objective of the third study was to replicate the findings from the first two studies by employing an alternative task of complex decision making with uncertain outcomes. A delay discounting task was chosen to examine whether people in a hot state (i.e., being hungry) would be better able to make advantageous choices compared to people who were not hungry. This study also examines in what way hot states affects risk taking and perceived reward value as two important components of decision making in hot states. We hypothesize that hot states not necessarily lead to taking more risks as the common explanation of decision making under hot conditions would predict. We also hypothesize that hot states may result in increased motivation to get the desired reward (reward perception) but not to the extent that it compromises beneficial decisions.

### Method

#### Participants

Forty-six university students (14 men, 32 women; mean age 20.8 years) with normal weight to slight overweight (BMI, *M* = 21.62, *SD* = 2.46; range 16.60–27.44) were recruited and participated in exchange for money (3€) or course credit. One participant with a BMI of 36.05 which is >4 SD from the mean was excluded from all analyses.

#### Ethics statement

See Study 1. All participants provided written informed consent.

### Procedure and Materials

#### Hunger manipulation

The same hunger manipulation as in Study 1 was employed.

#### Assessment of complex decision making

The main dependent variable was a delay discounting task assessing the tendency to favor a delayed but larger monetary reward over an immediate smaller reward in a series of 27 hypothetical binary choices with varying delays (today vs. 7–186 days). Participants were presented with 27 questions requiring them to choose between a smaller and a larger amount of money which would be available to them after either a short while or a long while (e.g., “Would you prefer €27 today, or €50 in 21 days?”), following a previously established format developed by Kirby et al. [24] The dependent variable is the hyperbolic discount parameter *k* at indifference between the smaller immediate reward and the larger delayed reward (theoretically ranging from.00016 to.25), calculated for small (*k*_small_), medium (*k*_medium_), and large (*k*_large_) rewards. Smaller *k* scores represent less impulsive choices as future rewards are less devaluated. The large series of decisions about varying amounts of money within different time frames makes the delay discounting task complex as it impossible to determine exactly the most profitable scenario.

#### Assessment of risk taking and reward value

To address the question whether hunger would affect risk taking, we assessed performance on the Balloon Analogue Risk Task (BART). [33] The BART measures explicit willingness to risk loss by instructing participants that they can earn money by inflating a balloon but that they will lose the money earned so far when the balloon is pumped past its explosion point. The dependent variable is the average number of pumps excluding balloons that exploded, reflecting a tendency to continue with balloon inflation despite the risk of losing the money already won on that trial. [33].

To address the question whether hunger would affect reward perception, size perception of food, monetary objects, and a neutral object was assessed as an indirect measure of motivation to attain a reward that corresponds with a person's needs (e.g., obtaining food when hungry).

Size estimates of food (cookie), money (coin) and a neutral object (circle) allow for determining whether participants perceive a greater rewarding value of objects of desire (food or money) but not for neutral objects in the hot condition compared to the cool condition, as we would expect. Functional perception research suggests that objects that are means for reaching a goal are perceived as larger, thereby facilitating the ease with which such an object can be identified in the environment and thus increasing the likelihood of using the object for attaining goals. [34] We used an established procedure that requires participants to estimate the size of an object (in centimeters with two decimals) as it was presented on a computer screen. [35], [36] Size estimates also allow for examining the alternative explanation that hunger affects delay discounting because participants were more concerned about getting food than about earning money. Due to data recording failures, size perception measures were only available for 35 participants.

#### Other variables

To rule out the possibility that differences between conditions in delay discounting tasks were due do individual differences in impulsivity, the BIS/BAS scale was administered. [37] The Behavioral Inhibition System/Behavioral Activation System (BIS/BAS) scales involve 20 items with a response format ranging from 1 (completely disagree) to 4 (completely agree). A BIS score (7 items) and a total BAS (13 items) were computed, with Cronbach's alpha's of.83 and.79 respectively. Finally, demographic information was collected by asking participants about age, gender, weight and length. The latter two variables were used to compute BMI.

### Results

#### Randomization check

We observed no significant differences between conditions in terms of age, *F*(1, 44) = 2.88, *p* = .10, gender, BMI, or BIS, all *F*s <1, or BAS, *F*(1, 44) = 1.27, *p* = .27, indicating that randomization was successful.

#### Manipulation check

An ANOVA with condition as the independent variable and hunger as the dependent variable was conducted to test the validity of the hunger manipulation, yielding a significant effect, *F*(1, 44) = 53.68, *p*<.001, p*η* ^2^ = .55. As expected, participants in the hot (hunger) condition reported more hunger (*M* = 3.08, *SD* = .90) than those in the cool (sated) condition (*M* = .96, *SD* = 1.07).

#### Main analysis

Before conducting the main analyses, we examined correlations of the dependent variables with the BIS/BAS scales. These correlations were not significant (all *r'*s <.25, *p'*s >.09). These scales were therefore not included as a covariate. BMI was not significantly correlated with performance on the delay discounting task (*r*’s *<.24, p*’s >.10), and therefore not considered as a covariate. Non-parametric tests of the effect of condition on the skewed dependent variable *k* revealed a significant effect on *k*_large_ (*p*<.05), but not on *k*_medium_ or *k*_small_ (*p'*s >.25). Participants in the hot (hunger) condition reported lower discounting rates for large monetary rewards (*Md* = .004, *SD* = .015) than participants in the cool (sated) condition (*Md* = .010, *SD* = .013), showing that participants in a hot state were better able to appreciate big rewards that they needed to wait for.

#### Analysis of risk taking

To examine risk taking, an ANOVA with condition as the independent variable and BART performance as the dependent variable revealed no significant effect, *F* <1, demonstrating that participants in the hot (hungry) condition did not take more risks in pursuing a reward than participants in the cool (sated) condition.

#### Analysis of reward value

To examine reward perception, size perceptions of objects were analyzed with a repeated measures ANOVA with size perceptions of object type (cookies vs. coins vs. circle) as a within-subjects factor and condition (hot vs. cool as a between-subjects factor. Two participants who reported size estimates of coins (*N* = 1) or circles (*N* = 1) >3 SDs from the mean were excluded from the analysis. The analysis revealed a marginally significant main effect of condition, *F*(1, 32) = 3.98, *p* = .055, p*η* ^2^ = .11, with participants in the hot condition perceiving objects as bigger (*M* = 9.05, *SD* = 3.50) than those in the cool condition (*M* = 6.96, *SD* = 2.58). Significant effects for object type or the interaction between condition and object type were absent, *F*s <1. These results demonstrate that a hot state (hunger) did affect size perception of food, money and a neutral object to the same extent, although this effect was only marginally significant. Objects were in general estimated as bigger under conditions of hunger than under sated conditions, showing that (reward) perception is increased in a hot state while ruling out the alternative explanation that participants in the hunger condition were simply more interested in food rewards and less interested in monetary rewards compared to sated participants because of being hungry (Table 3).

**Table 3:** Study 3: Delay discounting and size perception as a function of hungry vs. sated state.

	Delay Discounting	Size Perception
	Md (SD)	M (SD)
Sated	.010 (.013)	6.96 (2.58)
Hungry	.004 (.015)	9.05 (3.50)

### Discussion

Study 3 provided further evidence that the hot state of hunger promoted rather than compromised complex decisions with uncertain outcomes that are advantageous in the long run as hungry participants were better able to resist (hypothetical) choices that brought immediate big (but not medium or small) rewards but were ultimately disadvantageous. Hunger did not affect BART performance, demonstrating that hungry people do not take more risk to jeopardize an amount of money to win a larger amount. Importantly, similar risk taking tendencies across both conditions were observed despite the perception of increased rewarding value of food and money in the hot condition, thus showing that the hot state of hunger increases motivation to get a reward but not at all prices. Similar size perceptions of food and monetary rewards also ruled out the alternative explanation that hungry participants did not care for money because of being more interested in food. Contrary to our expectations, the hot state of hunger also resulted in greater size estimations of a neutral object, challenging the notion that size perception relates to objects with a rewarding value only.

## General Discussion

This series of studies set out to test the hypothesis that hot states may benefit, rather than compromise, advantageous decision making insofar it concerns complex decisions with uncertain outcomes. Based on the notion that intuition and emotions may improve this specific category of decisions [10], [12], [13], we argued that hot states, which are known to make people more reliant on their feelings, improve their decisions. This assumption follows from theories on intuitive decision making but so far has not been tested explicitly by directly manipulating hot states. Our findings lend credit to these expectations: people who were moderately hungry or had a moderate appetite, compared to people who were satiated or had a lower appetite, made more advantageous decisions as witnessed by their performance on the IGT (Studies 1 and 2) and a delay discounting task (Study 3). These findings were obtained for both visceral (Studies 1 and 3) and non-visceral (Study 2) manipulations of a hot state. Importantly, Study 3 also revealed that a hot state (resulting from hunger or appetite) did not affect willingness to take risks in spite of the perception of an increased rewarding value of desired objects (food and money) as well as a neutral object, although the latter finding was unexpected. These findings speak directly to the mechanism involved in complex decision making under uncertain conditions. Typically, strategic decision making in complex situations without being certain what these decisions bring in the future may be conceived of as a trade-off between risk and reward, as exemplified in the IGT presenting people with decks of cards either involving big rewards but also a higher chance of loss or small rewards that are accompanied by lower chances of loss. In order to make decisions that are advantageous in the long run people thus must recognize the risk of loss when being tempted by a bigger reward. Our findings show that people in a hot state are better able to do so, as witnessed by their capability to make advantageous decisions (assessed by the IGT or a delay discounting task), while perceiving larger rewards (size perception task) but not taking more risks (BART performance). It has been demonstrated in many studies employing the IGT in clinical samples (with deficits in emotion processing) that not being able to use one's emotions for recognizing risk and resisting decisions that involve huge but risky rewards compromises complex decision making in uncertain conditions. [14], [15], [16], [17] However, it has not been examined previously that manipulating hot states in normal people without emotion processing deficits improves such decisions and has straightforward beneficial effects, presumably by making people rely more on their intuition and emotion.

An intriguing result from our research was the unexpected finding that people in a hot state do not only perceive those objects as bigger that are generally (money) or specifically (food when one is hungry) regarded as rewarding, but also the neutral object of a circle. As stated previously, functional perception theory [34] assumes that objects that are means for reaching a goal are perceived as larger, thereby facilitating the ease with which such an object can be identified in the environment and thus increasing the likelihood of using the object for attaining goals. It is unlikely that the neutral object of a circle would meet the criterion of being perceived as a means to attain a goal - although we cannot be rule out the possibility that participants in our studies perceived the circle as a means of engaging in a playful activity such as puzzle solving. However, it seems more likely that a hot state like hunger affects the perception of all kinds of objects and not only those that are functional for goal attainment. Until now, the effect of visceral states on size perception has rarely been addressed and future research should address these intriguing results in more detail. [38].

Together, these studies for the first time provide suggestive evidence that hot states improve complex decision making under uncertain conditions, lending support to our assumption that being able to recognize and use one's emotions benefits complex decisions. Apparently, our findings stand in sharp contrast with previous studies showing that hot states in general and visceral drives in particular compromise decision making. These studies generally assume that hot states make people more impulsive and disregard the risks of a behavior that seem so evident under cooler conditions. However, most studies so far either tested these assumptions in samples with impulsive pathology or used simple decision tasks that allowed for a straightforward comparison of the options involved. Also, previous studies did not manipulate hot states directly but, for example, compared the virtual versus tangible presence of cookies. [5] Our findings show that under the typical hot condition of hunger or appetite, an increased willingness to take risks is absent, even when an increased motivation for getting the reward is present.

We argue that these benefits from being in a hot state result from a greater reliance on emotions that allow for a better recognition of risks that go hand in hand with big rewards, as implied by Damasio's research on anticipated feelings as conditioned emotions. [12], [14] The present series of studies did not examine directly whether the beneficial effects of a hot state on complex decision making result from more reliance on one's emotions. However, previous studies in people with emotion processing deficits [15, 16. 17] give credit to this interpretation, as does our finding that hot states do not necessarily generate more risk taking. This would imply that insofar hot states make people more impulsive, impulsivity means that they act swift and without explicit deliberation. Such an account of the beneficial effect of hot states on complex decision making make sense when considering that many real life decisions do not allow for extended and deliberate reasoning because long-term effects of these decisions are difficult to predict. Notwithstanding this, future research should examine to what extent hot states make people rely on their emotions and whether reliance on one's emotions is the causal factor in promoting good decisions.

Our findings bear important implications for theorizing about the role of hot states in decision making. It may be, as suggested in the foregoing, that hot states in general, and hunger and appetite in particular, do not necessarily make people more impulsive but rather make them rely more on their gut feeling which benefits complex decisions with uncertain outcomes. Alternatively, it may be that hot states do increase impulsivity but that impulsivity is not necessarily bad. Such a conceptualization of good' impulsivity aligns with recent notions that negative consequences are not inherent in impulsive behavior. Being in an impulsive state entails that people are more inclined to make decisions quickly with little or no deliberation which may turn out either favorable or unfavorable depending on the demands of the situation. [39], [40] Adopting the view that impulsivity implies acting swiftly means that impulsivity brings an advantage as in a greater tendency to rely on emotions when confronted with the complex self-regulation dilemma of choosing between small immediate benefit versus delayed but larger benefit. This line of reasoning concords with recent critical notions about dual-system accounts of behavioral regulation, distinguishing between reflective (rational and cool) and reflexive (emotional and hot) systems. [19] Typically, dual-systems accounts conceive of the reflective system as being responsible for adaptive behavior in accordance with long-term goals and the reflexive system as being responsible for an impulsive breakdown that accounts for abandoning long-term goals, thus equating the process (reflective vs. reflexive) with the outcome (adaptive vs. non-adaptive. [3] However, recent research challenges this sharp distinction by showing evidence indicating that impulsive states can sometimes generate adaptive behavior. [20], [41], [42] By the same token, it has also been shown that reflective processes may be required to engage in bad' behavior, such as overcoming the initially aversive taste of alcohol or nicotine [43] or deliberate reasoning to find justifications for otherwise forbidden indulgent behavior [44]. Our finding that hot states promote advantageous decision making thus contributes to novel theorizing about impulses that were hitherto considered as compromising adaptive behavior.

### Limitations and future directions

We manipulated hunger and appetite to create typical hot states that are considered to induce impulsive behavior. Humans, like other animals, are evolved to get food and patience may be life threatening in case of hunger. [45] Moreover, food and money have been used in previous studies as salient and strong rewards that can influence decision making. [7] Future research should examine whether our observations hold for other kinds of hot states, either visceral (e.g., thirst, pain, sexual desire) or non-visceral (emotions), and for other types of rewards. In addition, it is important to determine at what level hot states may become dysfunctional. Participants in our studies who experienced benefit from being hungry reported only moderate levels of hunger (average scores of about 3 to 4 on a 5-point scale), which makes sense considering that extremely hungry participants would have been preoccupied with getting food which would probably have hampered their decisions (cf., [46] for similar reasoning in case of urination urgency). We thus hasten to add a cautionary notion that our findings may only hold for moderate levels of hunger or appetite rather than extreme levels. Another limitation relates to the specific sample under study. We recruited small samples of young and healthy university students, which may hamper the generalizability of our findings. Although we were able to reveal consistent patterns of results across three studies, future research should consider bigger samples that are more representative of the population and include older people with more diverse educational backgrounds. Finally, the generalizability of our findings should be addressed by including outcomes that involve decisions with more serious implications than the hypothetical rewards that are typically used in IGT and delay discounting tasks. Although the IGT is generally regarded as a task that captures the uncertainties that are characteristic of real life decision making [22], the somewhat artificial conditions under which the task is administered strongly require corroboration with more ecologically valid decision tasks or decision tasks outside the lab. Notwithstanding these limitations, our research demonstrates that an empty stomach increases our gut feeling for doing what is beneficial in the long run.

## References

[1] Hofmann W, Baumeister RF, Förster G, Vohs KD (2012) Everyday temptations: An experience sampling study of desire, conflict, and self-control. J Pers Soc Psych 102: 1318–1335. 10.1037/a0026545 22149456

[2] Loewenstein G (1996) Out of control: Visceral influences on behavior. Org Beh Hum Dec Proc 65: 272–292.

[3] Metcalfe J, Mischel W (1999) A hot/cool-system analysis of delay of gratification: Dynamics of willpower. Psych Rev 106: 3–19. 10.1037/0033-295x.106.1.3 10197361

[4] Ariely D, Loewenstein G (2006) The heat of the moment: The effect of sexual arousal on sexual decision making. J Beh Dec Making 19: 87–98.

[5] Ditto PH, Pizarro DA, Epstein EB, Jacobson JA, McDonald TK (2006) Visceral influences on risk-taking behavior. J Beh Dec Making 19: 99–113.

[6] Nordgren LF, Van der Pligt J, Van Harreveld F (2008) The instability of health cognitions: Visceral states influence self-efficacy and related health beliefs. Health Psych 27: 722–727. 10.1037/0278-6133.27.6.722 19025267

[7] Briers B, Pandelaere M, Dewitte S, Warlop L (2006) Hungry for money: The desire for caloric resources increases the desire for financial resources and vice versa. Psych Sci 17: 939–943. 10.1111/j.1467-9280.2006.01808.x 17176423

[8] Van den Bergh B, Dewitte S, Warlop L (2008) Bikinis instigate generalized impatience in intertemporal choice. J Cons Res 35: 85–97.

[9] Bechara A, Tranel D, Damasio H (2000) Characterization of the decision-making deficit of patients with ventromedial prefrontal cortex lesions. Brain 123: 2189–2202. 11050020 10.1093/brain/123.11.2189

[10] Dijksterhuis A, Nordgren LF (2006) A theory of unconscious thought. Persp Psych Sci 1: 95–109. 10.1111/j.1745-6916.2006.00007.x 26151465

[11] Nordgren LF, Dijksterhuis AP (2009) The devil is in the deliberation: Thinking too much reduces preference consistency. J Cons Res 36: 39–46.

[12] Damasio AR (1994) Descartes' error: Emotion, reason, and the human brain. New York: Grosset/Putnam Books.

[13] Loewenstein G, Weber EU, Hsee CK, Welch N (2001) Risks as feelings. Psych Bull 127: 267–286. 10.1037/0033-2909.127.2.267 11316014

[14] Bechara A, Damasio H, Tranel D, Damasio AR (1997) Deciding advantageously before knowing the advantageous strategy. Sci 275: 1293–1295. 10.1126/science.275.5304.1293 9036851

[15] Crone EA, Vendel I, Van der Molen MW (2003) Decision-making in disinhibited adolescents and adults: insensitivity to future consequences or driven by immediate reward. Pers Ind Diff 35: 1625–1641.

[16] Davis C, Patte K, Tweed S, Curtis C (2007) Personality traits associated with decision-making deficits. Pers Indiv Diff 42: 279–290.

[17] Zermatten A, Vanderlinden M, d'Acremont M, Jermann F, Bechara A (2005) Impulsivity and decision making. J Nerv Ment Dis 193: 647–650. 16208159 10.1097/01.nmd.0000180777.41295.65

[18] Seo M, Barrett LF (2007) Being emotional during decision making – good or bad? An empirical investigation. Acad Man J 50: 923–940. 10.5465/amj.2007.26279217 PMC2361392 18449361

[19] De Witt Huberts JC, Evers C, De Ridder DTD (2014) ‘Because I am worth it': A theoretical framework and empirical review of a justification-based account of self-regulation failure. Pers Soc Psych Rev 18: 119–138. 10.1177/1088868313507533 24214148

[20] Salmon SJ, Fennis BM, De Ridder DTD, Adriaanse MA, De Vet E (2014) Health on impulse: When low self-control promotes healthy food choices. Health Psych 33: 103–109. 10.1037/a0031785 23477580

[21] Bechara A, Damasio AR, Damasio H, Anderson SW (1994) Insensitivity to future consequences following damage to human prefrontal cortex. Cogn 50: 7–15. 10.1016/0010-0277(94)90018-3 8039375

[22] Toplak ME, Sorge GB, Benoit A, West RF, Stanovich KE (2010) Decision-making and cognitive abilities: A review of associations between Iowa Gambling Task performance, executive functions, and intelligence. Clin Psych Rev 30: 562–581. 10.1016/j.cpr.2010.04.002 20457481

[23] Turnbull OH, Evans CEY, Bunce A, Carzolio B, O’Connor J (2005) Emotion-based learning and central executive resources: An investigation of intuition and the Iowa Gambling Task. Brain Cogn 57: 244–247. 15780457 10.1016/j.bandc.2004.08.053

[24] Kirby KN, Petry NM, Bickel WK (1999) Heroin addicts have higher discount rates for delayed rewards than non-drug-using controls. J Exp Psych Gen 128: 78–87. 10.1037//0096-3445.128.1.78 10100392

[25] Duckworth AL, Kern ML (2011) A meta-analysis of the convergent validity of self-control measures. J Res Pers 45: 259–268. 21643479 10.1016/j.jrp.2011.02.004 PMC3105910

[26] WMO (2012) *Central Committee on Research Involving Human Subjects* Available: http://www.ccmo-online.nl. Accessed 25 January 2012.

[27] De Ridder DTD, Ouwehand C, Stok FM, Aarts FJ (2011) Hot or not: Visceral influences on coping planning for weight loss attempts. Psych Health 26: 501–516. 10.1080/08870440903564332 20936566

[28] Kuijer R, De Ridder DTD, Ouwehand C, Houx B, Van den Bos R (2008) Dieting as a case of behavioral decision making. Does self-control matter? App 51: 506–511. 10.1016/j.appet.2008.03.014 18479777

[29] Dunn BD, Dalgleish T, Lawrence AD (2006) The somatic marker hypothesis: A critical evaluation. Neurosci Biobeh Rev 30: 239–271. 10.1016/j.neubiorev.2005.07.001 16197997

[30] Patton JH, Stanford MS, Barratt ES (1995) Factor structure of the Barratt Impulsiveness Scale. J Clin Psych 51: 768–774. 10.1002/1097-4679(199511)51:6<768::aid-jclp2270510607>3.0.co;2-1 8778124

[31] Maridakis V, Herring MP, O'Connor PJ (2009) Sensitivity to change in cognitive performance and mood measures of energy and fatigue in response to differing doses of caffeine and breakfast. Int J Neurosci 119: 975–994. 19466633 10.1080/00207450802333995

[32] Tangney JP, Baumeister RF, Boone AL (2004) High self-control predicts good adjustment, less pathology, better grades, and interpersonal success. J Pers 72: 271–322. 15016066 10.1111/j.0022-3506.2004.00263.x

[33] Lejuez CW, Read JP, Kahler CW, Richards JB, Ramsey SE, et al (2002) Evaluation of a behavioral measure of risk taking: The Balloon Analogue Risk Task (BART). J Exp Psych Appl 8: 75–84. 10.1037//1076-898x.8.2.75 12075692

[34] Bruner JS, Goodman CC (1947) Value and need as organizing factors in perception. J Abn Soc Psych 42: 33–44. 10.1037/h0058484 20285707

[35] Van Koningsbruggen GM, Stroebe W, Aarts H (2011) Through the eyes of dieters: Biased size perception of food following tempting food primes. J Exp Soc Psych 47: 293–299.

[36] Veltkamp M, Aarts H, Custers R (2008) Perception in the service of goal pursuit: Motivation to attain goals enhances the perceived size of goal-instrumental objects. Soc Cogn 26: 720–736.

[37] Carver CS, White TL (1994) Behavioral inhibition, behavioral activation, and affective responses to impending reward and punishment: The BIS/BAS scales. J Pers Soc Psych. 67: 319–333.

[38] Den Daas C, Häfner M, De Wit J (2013) Sizing opportunity: Biases in estimates of goal-relevant objects depend on goal congruence. Soc Psych Pers Sci 4: 361–367.

[39] Dickman SJ (1990) Functional and dysfunctional impulsivity: Personality and cognitive correlates. J Pers Soc Psych 58: 95–102. 10.1037//0022-3514.58.1.95 2308076

[40] Kacelnik A (2003) The evolution of patience. In Loewenstein G, Read D, Baumeister RF, editors. Time and decision. Economic and psychological perspectives on intertemporal choice. New York: Russell Sage Foundation, 115–138.

[41] Apfelbaum EP, Sommers SR (2009) Liberating effects of losing executive control. When regulatory strategies turn maladaptive. Psych Sci 20: 139–143. 10.1111/j.1467-9280.2009.02266.x 19170942

[42] Fennis BM, Janssen L Vohs KD (2009) Acts of benevolence: A limited resource account of compliance with charitable requests. J Cons Res 35: 906–924.

[43] Rawn CD, Vohs KD (2011) People use self-control to risk personal harm: An intra-personal dilemma. Pers Soc Psych Rev 15: 267–289. 10.1177/1088868310381084 20807858

[44] De Witt Huberts JC, Evers C, De Ridder DTD (2012) License to sin: Self-licensing as.

[45] Pinel JPJ, Assanand S, Lehman DR (2000) Hunger, eating, and ill health. Am Psych 55: 1105–1116. 10.1037//0003-066x.55.10.1105 11080830

[46] Tuk MA, Trampe D, Warlop L (2011) Inhibitory spillover: Increased urination urgency facilitates impulse control in unrelated domains. Psych Sci 22: 627–633. 10.1177/0956797611404901 21467548

